# The Effect of Serine Protease Inhibitors on Airway Inflammation in a Chronic Allergen-Induced Asthma Mouse Model

**DOI:** 10.1155/2014/879326

**Published:** 2014-08-07

**Authors:** Chih-Che Lin, Li-Jen Lin, Shulhn-Der Wang, Chung-Jen Chiang, Yun-Peng Chao, Joseph Lin, Shung-Te Kao

**Affiliations:** ^1^Graduate Institute of Chinese Medicine, China Medical University, Taichung 40402, Taiwan; ^2^School of Chinese Medicine, College of Chinese Medicine, China Medical University, Taichung 40402, Taiwan; ^3^School of Post-Baccalaureate Chinese Medicine, College of Chinese Medicine, China Medical University, Taichung 40402, Taiwan; ^4^Department of Medical Laboratory Science and Biotechnology, China Medical University, Taichung 40402, Taiwan; ^5^Department of Chemical Engineering, Feng Chia University, Taichung 40724, Taiwan; ^6^School of Medicine, Semmelweis University, Budapest 1085, Hungary; ^7^Department of Chinese Medicine, China Medical University Hospital, Taichung 40402, Taiwan

## Abstract

Serine protease inhibitors reportedly attenuated airway inflammation and had antioxidant in multiorgan. However, the effects of the serine protease inhibitors nafamostat mesilate (FUT), gabexate mesilate (FOY), and ulinastatin (UTI) on a long-term challenged mouse model of chronic asthma are unclear. BALB/c mice (6 mice/group) were intratracheally inoculated with five doses of *Dermatophagoides pteronyssinus* (Der p; 50 *μ*L, 1 mg/mL) at one-week intervals. Therapeutic doses of FUT (0.0625 mg/kg), FOY (20 mg/kg), or UTI (10,000 U/kg) were, respectively, injected intraperitoneally into these mice. Control mice received sterile PBS. At 3 days after the last challenge, mice were sacrificed to assess airway hyperresponsiveness (AHR), remodeling, and inflammation; lung histological features; and cytokine expression profiles. Compared with untreated controls, mice treated with FUT, FOY, and UTI had decreased AHR and goblet cell hyperplasia, decreased eosinophil and neutrophil infiltration, decreased Der p-induced IL-4 levels in serum and IL-5, IL-6, IL-13, and IL-17 levels in bronchoalveolar lavage fluid, and inhibited nuclear factor (NF)-*κ*B activity in lung tissues. The serine protease inhibitors FUT, FOY, and UTI have potential therapeutic benefits for treating asthma by downregulating Th2 cytokines and Th17 cell function and inhibiting NF-*κ*B activation in lung tissue.

## 1. Introduction

Asthma is a common chronic inflammatory disease of the airways and is characterized by intermittent attacks of breathlessness, airway hyperreactivity, wheezing, and cough in response to allergen exposure. The chronic inflammation in asthma is characterized by eosinophilic recruitment, airway hyperresponsiveness (AHR), goblet cell hyperplasia/metaplasia, epithelial hypertrophy/hyperplasia, mucus hypersecretion, collagen deposition, smooth muscle cell hypertrophy/hyperplasia, and subepithelial fibrosis [1, 2].

Activated pulmonary epithelial cells use ROS as a component of the intracellular signaling cascade induced subsequent CD4+ T cells activation [3]. CD4+ T cells, including Th1, Th2, Th17, and Treg cells, are active during different phases of bronchial asthma, and the expression of their various cellular products play different roles in lung inflammation [4]. Both extracellular endogenous proteases and exogenous proteases also play major roles in asthma pathophysiology. The serine protease tryptase, which is abundant in mast cell granules, is a potent protease-activated receptor-2 (PAR-2) activator [5]. Tryptase promotes human mast cell degranulation, mediates eosinophil and neutrophil migration, amplifies histamine's bronchoconstrictor effects on lung tissue, and stimulates airway fibroblast and smooth muscle cell growth [6, 7]. Furthermore, many aeroallergens associated with asthma, such as house dust mites and fungal allergens, are exogenous proteases. Two major dust mite antigens, Der p 3 and Der p 9, are serine proteases that can increase vascular permeability and detach epithelial cells by interacting with PAR-2 [8]. Therefore, targeting proteolytic activity with specific inhibitors may decrease protease-induced inflammatory processes.

Purified human urinary trypsin inhibitor (UTI), a serine protease inhibitor, has been widely used for patients with acute inflammatory disorders such as disseminated intravascular coagulation (DIC), shock, and pancreatitis [9]. Gabexate mesylate (FOY) and nafamostat mesilate (6-amidino-2-naphthyl p-guanidinobenzoate dimethane sulfonate; FUT) are nonantigenic synthetic serine protease inhibitors that have been used to treat pancreatitis and DIC and as anticoagulants for extracorporeal circulation with hemodialysis [10, 11]. In a previous report using an acute murine asthma model, we demonstrated that FUT and FOY inhibited antigen-induced airway eosinophilia, IgE production, and IL-4 and TNF-*α* levels and augmented IL-12 levels, a critical Th1 cytokine, in bronchoalveolar lavage fluid (BALF); however, UTI had no effect [12]. However, Yasui et al. reported that UTI may be a biomarker of asthma exacerbation in children [13]. Ishizaki et al. suggested that FUT inhibited airway eosinophilic inflammation and airway epithelial remodeling in an ovalbumin (OVA) murine model of allergic asthma [14]. However, the immunomodulatory effects of these synthetic and purified serum serine protease inhibitors in a Der p-induced chronic asthma model remain unknown.

Therefore, we investigated the potential therapeutic effects of the potent serine protease inhibitors FUT, FOY, and UTI on allergic airway inflammation and airway remodeling in a Der p-challenged murine model of chronic asthma.

## 2. Materials and Methods

### 2.1. Ethics Statement

Animal experiments were conducted in accordance with the principles outlined by the Institutional Animal Care and Use Committee of China Medical University. This study was granted an Affidavit of Approval of Animal Use protocol by China Medical University (IACUC no. 101-97-N). Mice were housed in microisolator cages (Laboratory Products, Inc., Maywood, NJ, USA) and were fed sterile food and water ad libitum in specific-pathogen free condition at the animal facilities of the China Medical University. Mice were sacrificed by i.p. injection of xylazine (200 *μ*g/mouse) and ketamine (2 mg/mouse).

### 2.2. Mice and Reagents

Specific pathogen-free, male, 6- to 8-week-old BALB/c mice were purchased from the National Laboratory Animal Center, Republic of China. Der p extract (1 g lyophilized whole body extract in ether; Allergon, Engelholm, Sweden) was dissolved in pyrogen-free isotonic saline, filtered through a 0.22 mm filter, and stored at −80°C until further use. LPS levels in the Der p preparations were <0.96 EU/mg of Der p (limulus amebocyte lysate test, E-Toxate; Sigma-Aldrich, St. Louis, MO). FUT (Futhen; Torii Pharmaceuticals Co, Chiba, Japan), FOY (Ono Pharmaceutical Co, Osaka, Japan), and UTI (Mochida Pharmaceuticals Co, Tokyo, Japan), which have been used clinically, were dissolved in sterile PBS before use.

### 2.3. Der p Challenge and Bronchoalveolar Lavage (BAL)

Each experiment included the following groups (*n* = 6/group) and mice were divided randomly. BALB/c mice were intratracheally inoculated with five doses of Der p (1 mg/mL, 50 *μ*L) at 1-week intervals. Mice were intraperitoneally (i.p.) injected with recommended therapeutic doses of FUT (0.0625 mg/kg), FOY (20 mg/kg), or UTI (10,000 U/kg) during intratracheal Der p challenge periods. In parallel experiments, naïve mice were intraperitoneally (i.p.) injected with sterile PBS and challenged with PBS. Mice were sacrificed 3 days after the last challenge, as reported previously [15]. The trachea was exposed and cannulated, and then BAL was performed with two 1 mL aliquots of saline. A total of 1.8 to 1.9 mL of BALF was consistently recovered with this technique. BALF samples were stored at −80°C until further assay. Differential cell counts were made using cytospin preparations (1 × 10^5^ cells/100 *μ*L of BALF) stained with Liu stain (Biotech, Taiwan) in a blinded manner after total leukocyte counting.

### 2.4. Invasive Measurement of Airway Resistance

On the day of the experiment, mice were weighed and anesthetized with sodium pentobarbital (60 mg/kg, i.p.). A mouse was tracheostomized using an 18G metal cannula. Respiratory system resistance (Rrs) and respiratory system elastance (Ers) were measured using a flow-type body plethysmograph connected via an endotracheal cannula to a* flexiVent* system (SCIREQ, Inc., Montreal, Canada) according to the manufacturer's protocol. Mice treated with or without protease inhibitors were sequentially exposed to increasing doses of nebulized methacholine in PBS: 0, 0.0625, 0.125, 0.25, 0.5, 1.0, 2.0, and 4.0 mg/mL (Sigma-Aldrich, St. Louis, MO).

### 2.5. Lung Tissue Histology

Paraffin-embedded lung tissue was cut into 5 *μ*m sections and stained with periodic acid–Schiff (PAS) stain. Light microscopy (400× magnification) was used for histological assessments. Goblet cell hyperplasia in the epithelial lining was scored as the percentage of goblet cells among epithelial cells [14]. To minimize sampling errors, a five-point scoring system (grades 0 to 4) was used: grade 0, no goblet cells; grade 1, <25% goblet cells; grade 2, 25–50% goblet cells; grade 3, 50–75%; and grade 4, ≥75%.

### 2.6. Collagen Analysis

Lung tissue (100 mg) from each group of mice was mechanically homogenized in liquid nitrogen and extracted in 2 mL of Hank's Balanced Salts Solution (HBSS). Collagen was quantified with a Sircol collagen assay kit (Biocolor, Belfast, UK).

### 2.7. Flow Cytometry Analysis

Monoclonal antibodies (mAbs) used included PE and/or FITC-conjugated anti-mouse CD4 (BD Pharmingen), FITC-conjugated anti-mouse CD8 (BD Pharmingen), Percp-conjugated anti-mouse CD3 (BD Pharmingen), and FITC-conjugated anti-mouse CD25. BALF cells (1 × 10^5^) were stained with an mAb for 30 min on ice, washed, and quantified using FACScan (Becton-Dickinson Immunocytometry System, San Jose, CA, USA).

### 2.8. Serum Der p-Specific IgG1 and IgG2a/2b and Total IgE

Serum samples for total IgE (1 : 2 in buffer) and Der p-specific IgG1 and IgG2a/2b (1 : 4 in buffer) were used for ELISA. Briefly, 96-well plates were coated with Der p (2 g/mL in 0.1 M Na_2_CO_3_/NaHCO_3_, pH 8.3). After overnight incubation at 4°C, the plates were washed and then duplicate serum samples were added for 2 h. After washing, biotin anti-mouse IgE antibody (2 *μ*g/mL; BD PharMingen) was added to wells for 1 h, followed by washing and adding a streptavidin-HRP conjugate (1 : 1000; BD PharMingen). The plates were washed and developed with a TMB microwell peroxidase substrate (Kirkegaard & Perry Laboratories, Gaithersburg, MD). Absorbance was read at OD_450_. Total IgE levels were determined from curves prepared using commercial mouse IgE standards. Der p-specific IgE, IgG1, and IgG2a/2b were detected using biotin anti-mouse IgE, IgG1, and IgG2a/2b antibodies (2 *μ*g/mL; BD PharMingen), with results expressed as OD_450_ values.

### 2.9. Cytokine ELISA

IL-4, IL-5, IL-6, IL-12, and IL-17 were measured with an ELISA Ready-SET-Go! Kit (eBioscience, San Diego, CA). IL-13 and INF-*γ* were measured using an ELISA DuoSet! Kit (R&D System, Abingdon, UK). Both assays were performed according to the manufacturers' protocols using standards supplied with the kits.

### 2.10. Quantitative Real-Time Polymerase Chain Reaction (qPCR)

Total RNA was extracted from lung tissue using Trizol reagent (Invitrogen Life Technologies) according to the manufacturer's protocol. Total RNA samples were reverse-transcribed to cDNA using a High Capacity cDNA Reverse Transcription Kit (Applied Biosystems, Foster City, CA, USA). qPCR was performed using 1 *μ*L of cDNA and a FastStart Universal SYBR Green Master kit (Roche). The following primer sets were used: *β*-actin, 5′-TGGAATCCTGTGGCATCCATGAAAC-3′ and 5′-TAAAACGCAGCTCAGTAACAGTCCG-3′; TGF-*β*1, 5′-GCGGACTACTATGCTAAAGATGT-3′ and 5′-GTTGTGTTGGTTGTAGAGGGGCA-3′; RANTES, 5′-GTACATCACCATGGCGTATG-3′ and 5′-TCTTCTCTGGGTTGGCACACA-3′; eotaxin, 5′-CCATCTGTCTCCCTCCACCATG-3′ and 5′-ATCCCACATCTCCTTTCATGCC-3′; GATA-3, 5′-GAAGGCATCCAGACCCGAAAC-3′ and 5′-ACCCATGGCGGTGACCATGC-3′; IL-6, 5′-CTGGTGACAACCACGGCCTTCCCTA-3 and 5-ATGCTTAGGCATAACGCACTAGGTA-3′; IL-13, 5′-CTGCAGTCCTGGCTCTCG-3′ and 5′-CTTTTCCGCTATGGCCACTG-3′; IL-17, 5′-AGATCTGGACGCGCAAACATGAG-3′ and 5′-GGGTCGTCGACGGGTCTCTGTTTAG-3′.

### 2.11. Nuclear Extracts and EMSA

Nuclear extracts were prepared from lung tissue according to the manufacturer's instructions (Panomics, Redwood). A LightShift Chemiluminescent EMSA Kit (PIERCE, Rockford) was used to detect NF-*κ*B expression.

### 2.12. Statistical Analysis

Results are expressed as means ± standard deviations. Student's *t*-test was used to compare the results between two groups. A *P* value of <0.05 was considered statistically significant.

## 3. Results

### 3.1. Protease Inhibitor Effects on Mouse Airway Resistance

**Figure 1:**
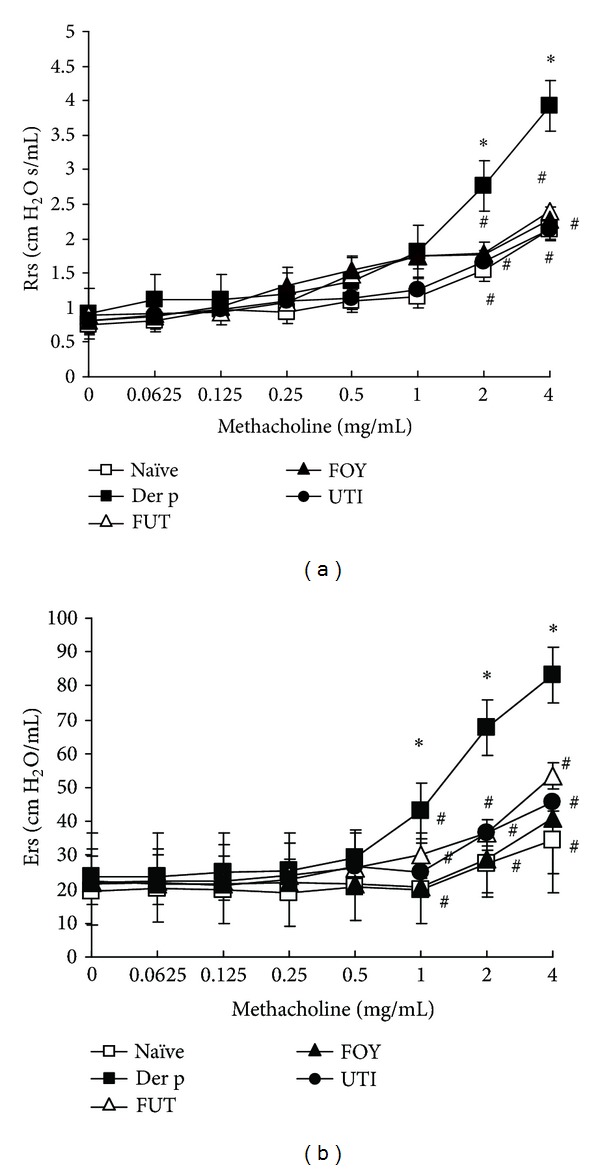
Effects of protease inhibitors on Der p-induced AHR. Rrs and Ers values were determined 3 days after the last Der p challenge using methacholine challenge tests (see Methods). Results are expressed as means ± SDs for 6 mice/group. *P < 0.05 versus naïve group; ^#^ *P* < 0.05 between Der p-treated groups.

We used repetitive Der p challenges to test the effects of three protease inhibitors (FUT, FOY, and UT1) on allergen-induced chronic airway inflammation in a mouse model. At 72 h after the last allergen challenge, all groups of mice were subjected to methacholine stimulation testing. Compared with the unchallenged naïve group, mice in the Der p-challenged group had markedly higher Rrs and Ers values. In the groups that received the protease inhibitors, the Rrs and Ers values were significantly decreased relative to those in the Der p group for methacholine doses ≥1.0 mg/mL (Figure 1).

### 3.2. Protease Inhibitor Effects on Der p-Induced Airway Inflammation

**Figure 2:**
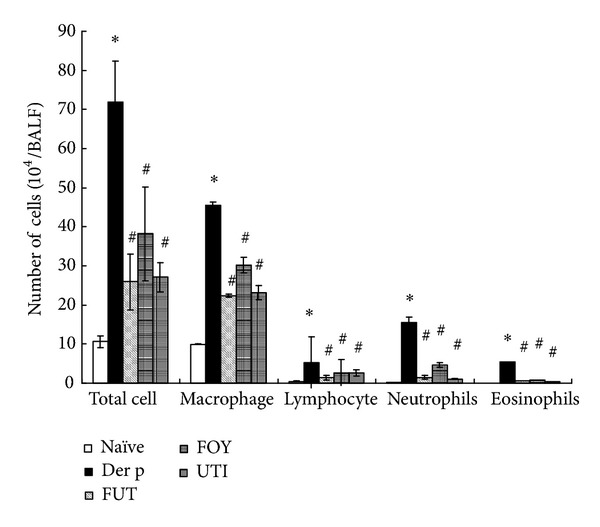
Effects of protease inhibitors on Der p-induced airway inflammatory cell infiltrates in BALF. Total cell numbers and cell differentials in BALF of BALB/c mice were determined at 72 h after the last Der p challenge, as described in Methods. Results are expressed as means ± SDs for 6 mice/group. *P < 0.05 versus naïve group; ^#^ *P* < 0.05 between Der p-treated groups.

BALB/c mice were intratracheally inoculated with five doses of Der p in PBS at 1-week intervals and sacrificed at 72 h after the last challenge. In the naïve unchallenged group, most cells in BALF were macrophages (Figure 2). However, the total number of cells, macrophages, neutrophils, lymphocytes, and eosinophils was markedly higher in mice exposed to Der p than naïve group, and it was significantly lower in the FUT, FOY, and UTI groups than in the Der p group.

### 3.3. Protease Inhibitors Attenuate Der p-Induced Lung Pathology

**Figure 3:**
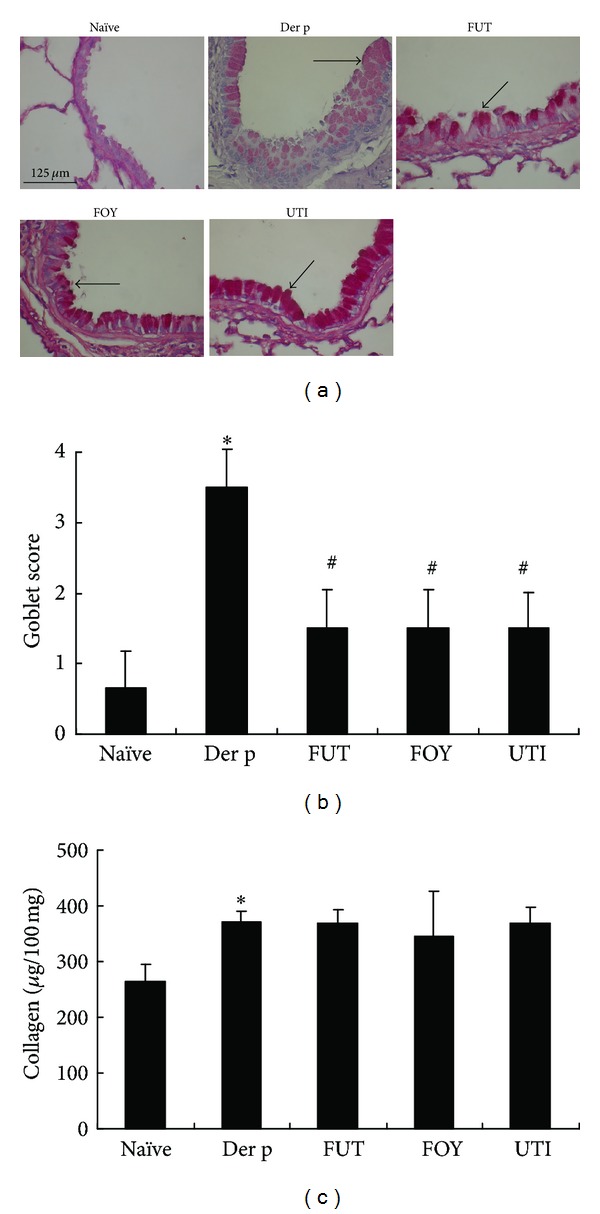
Effects of protease inhibitors on Der p-induced airway inflammation, goblet cell hyperplasia, mucus hypersecretion, and collagen deposition in mouse lung tissues. (a) Goblet cells (PAS; original magnification, ×400) in airway epithelial cells and (b) goblet cell hyperplasia was scored on a 5-point scale (0–4). (c) Collagen contents in lung tissue results are expressed as means ± SD's for 6 mice/group. *P < 0.05 versus naïve group; ^#^ *P* < 0.05 between Der p-treated groups.

The characteristic features of asthmatic airways include inflammation, hyperplastic goblet cells, mucus secretion, and collagen deposition. The lungs of mice were histologically examined at 72 h after the last antigen challenge (Figure 3(a)). Relative to the naïve group, histological lung tissue sections from Der p-challenged mice exhibited increased airway inflammation, matrix deposition in subepithelial regions, and hyperplastic goblet cells (Figure 3(b)). In contrast, mice treated with serine protease inhibitors showed significantly decreased airway inflammation, fewer PAS-positive cells, and less hyperplastic goblet cells compared with the Der p group. However, there was no significant difference in collagen deposition levels between the FUT, FOY, and UTI groups and Der p group (Figure 3(c)).

### 3.4. Protease Inhibitor Effects on T-cell Subsets in BALF and Serum Antibody Levels

**Figure 4:**
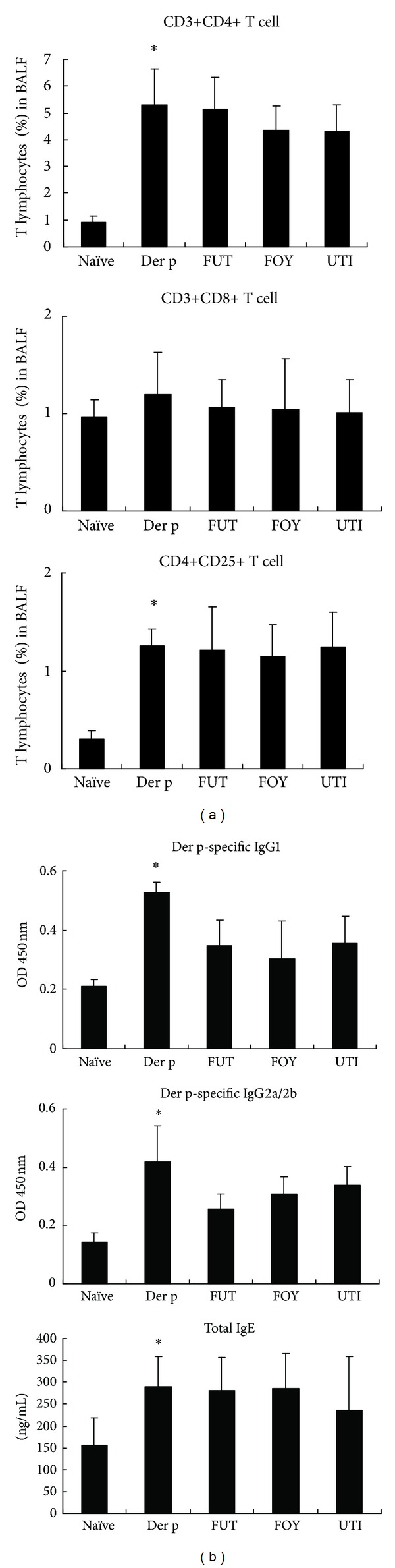
Effects of protease inhibitors on T-cell subsets in BALF and serum antibodies. (a) The percentage of CD3+/CD4+, CD3+/CD8+, and CD4+/CD25+ lymphocytes was determined by flow cytometry. (b) Total IgE and Der p-specific IgG1 and IgG2a/2b levels were determined by ELISA. Results are expressed as means ± SD's for 6 mice/group. *P < 0.05 versus naïve group; ^#^ *P* < 0.05 between Der p-treated groups.

Protease inhibitor effects on T-cell subsets in BALF were determined by flow cytometry and monoclonal antibodies for CD3+, CD4+, CD8+, and CD25+ (Figure 4(a)). Relative to the naïve group, there were significant increases in the percentage of CD3+/CD4+ and CD4+/CD25+ T-cells in the Der p group. However, there were no significant differences in the percentages of CD3+/CD4+ and CD4+/CD25+ lymphocytes between the FUT, FOY, and UTI groups and the Der p group. The percentage of CD3+/CD8+ T-cells in BALF was not different among the five groups.

To assess the effects of protease inhibitors on humoral immune response status of repetitively Der p-challenged mice, serum samples were assayed for Der p-specific IgG1 and IgG2a/2b and total IgE levels (Figure 4(b)). Compared with naïve mice, serum Der p-specific IgG1 and IgG2a/2b and total IgE levels were significantly increased in the Der p group. However, there was no significant difference in the levels of these antibodies between the FUT, FOY, and UTI groups and Der p group.

### 3.5. Protease Inhibitors Decrease Proinflammatory and Th2 Cytokines in BALF and Serum

**Figure 5:**
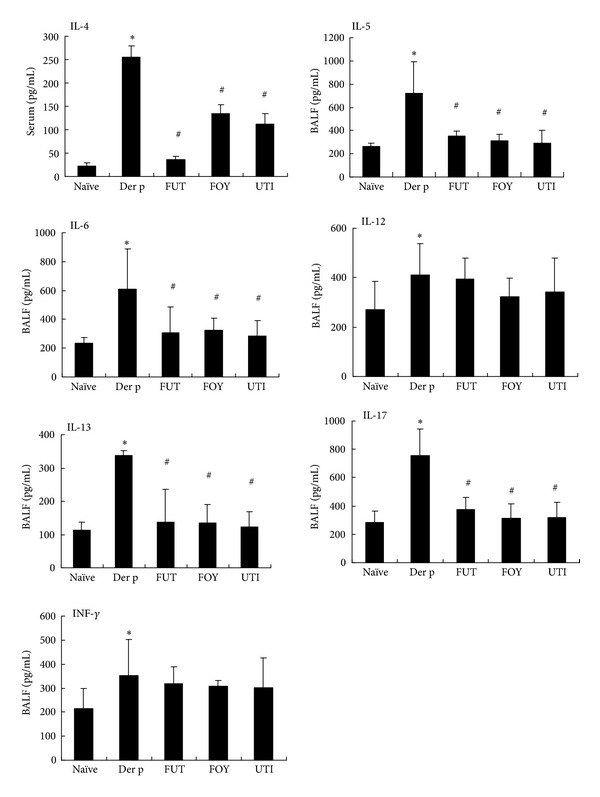
Effects of protease inhibitors on IL-5, IL-6, IL-13, and IL-17 levels in BALF and IL-4 levels in serum. Results are expressed as means ± SDs for 6 mice/group. *P < 0.05 versus naïve group; ^#^ *P* < 0.05 between Der p-treated groups.

To determine if these protease inhibitors had any effects on T-cell-mediated responses, we assayed for T-cell cytokines in BALF and serum. As shown in Figure 5, compared with Der p treatment, FUT, FOY, and UTI treatment significantly decreased the levels of IL-4 in serum and those of IL-5, IL-6, IL-13, and IL-17 in BALF. However, IL-12 and IFN-*γ* levels in BALF were not affected by FUT, FOY, and UTI treatment.

### 3.6. Protease Inhibitors Regulate Der p-Induced Proinflammatory Cytokine and Chemokine Gene Expression in Lung Tissue

**Figure 6:**
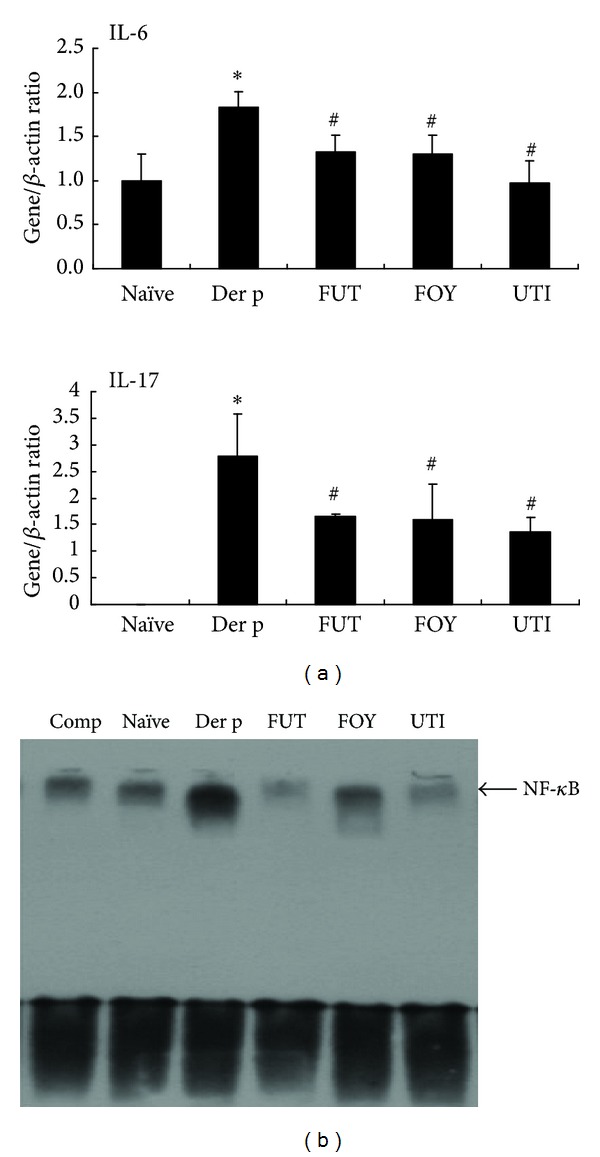
Effects of protease inhibitors on IL-6 and IL-17A gene expression and NF-*κ*B activity in lung tissue. (a) Relative IL-6 and IL-17A mRNA expression in lung tissue. Results are expressed as means ± SDs for 6 mice/group. *P < 0.05 versus naïve group; ^#^ *P* < 0.05 between Der p-treated groups and (b) EMSA showed that all three serine protein inhibitors decreased NF-*κ*B activation in lung tissue after Der p stimulation. The arrow indicates the specific DNA-probe complex.

Compared with the Der p group, FUT, FOY, and UTI treatment resulted in significantly lower levels of IL-6 mRNA and IL-17A mRNA expression (relative to *β*-actin mRNA) in lung tissue (Figure 6(a)). The relative mRNA levels of other genes, including those for TGF-*β*1, RANTES, eotaxin, GATA-3, and IL-13, were not significantly different in the FUT, FOY, and UTI groups compared with those in the Der p group (data not shown).

### 3.7. Protease Inhibitors Suppress NF-*κ*B Activation in Der p-Challenged Lung Tissues

The transcription factor nuclear factor (NF)-*κ*B plays an essential regulatory role in the production of proinflammatory cytokines such as TNF-*α*, IL-6, and IL-1. There are therapeutic benefits of inhibiting NF-*κ*B activity in inflammatory conditions [16].  Figure 6(b) shows the results of NF-*κ*B activation in whole lungs from the five groups of mice using an electrophoretic mobility shift assay (EMSA). NF-*κ*B-specific DNA-protein binding activity was markedly increased in lung tissues in the Der p group. In contrast, mice treated with FUT, FOY, and UTI showed markedly inhibited NF-*κ*B activity compared with those treated with Der p.

## 4. Discussion

Studies using murine experimental models have significantly contributed to our understanding of allergic inflammatory mechanisms that underlie asthma. However, models involving short-term, high-level exposure of sensitized animals to antigen have severe limitations for investigating the pathogenesis of lesions that develop in chronic asthma [1]. We previously showed that repetitive challenges by intratracheal exposure to house dust mites, without adjuvant therapy, resulted in chronic pulmonary inflammation. Mice used in this model exhibited several symptoms that were similar to those observed in humans with chronic asthma, including high allergen-specific IgE levels; pulmonary eosinophilia and AHR; and increased IL-5, IL-13, IL-17, and IFN-*γ* levels in BALF; increased co-stimulatory B7.2 expression on BALF cells; and features of airway remodeling, including collagen deposition and goblet cell hyperplasia [17, 18]. In this study, we assessed the possible protective effects of FUT, FOY, and UTI administered during the sensitization period in a murine model of chronic asthma.

Airway hyperresponsiveness (AHR) is a key pathophysiological feature of asthma. Tracheal pressure (Ptr) has revealed much about the mechanisms of AHR in asthma, as well as the mechanisms of action of pharmacotherapy used to inhibit AHR. Ptr is combined by airway flow pressure and airway elastance pressure [19]. The Rrs and Ers are two important coefficients for tracheal pressure (Ptr) according to an equation of respiratory system motion: Ptr = Ers × *V*(*t*) + Rrs × *V*′(*t*), where *t* is time, *V* is lung volume change, and *V*′ is airflow. When allergen enter the airway can induce airway obstruction, it increases Rrs and Ers levels for airway resistance in asthma [20, 21]. In this study, treatment with the three serine protease inhibitors suppressed Rrs and Ers values and airway inflammation and remodeling. These serine protease inhibitors had anti-inflammatory effects, as reflected by the decrease in the total number of cells and the percentage of macrophages, neutrophils, lymphocytes, and eosinophils in BALF relative to those in the Der p group. Moreover, these inhibitors also decreased airway inflammation and the number of hyperplastic goblet cells, as shown by lung histology, although they did not alter collagen deposition. Therefore, the suppressive effects of FUT, FOY, and UTI on AHR may be associated with decreased airway inflammation, eosinophil infiltration, and goblet cell hyperplasia in airway remodeling. However, these serine proteases had no significant effects on the percentage of CD3+CD4+, CD3+CD8+, and CD25+CD4+ T-cell subsets in BALF or on different antibody isotypes in serum.

Th2 cytokines such as IL-4, IL-5, and IL-13 play an important role in the pathophysiology of asthma, including AHR and airway wall remodeling [22]. In asthma pathogenesis, IL-4 is an upstream cytokine that regulates allergic inflammation by promoting Th2 cell differentiation [23]. IL-5 is specific for eosinophil terminal differentiation, growth, and survival [24, 25]. IL-4 and IL-13 induce mucus hypersecretion and contribute to an increase in AHR together with IL-5 and airway remodeling together with TGF-*β* and IL-6 [26]. In this study, we showed that serine protease inhibitors decreased the secretion of IL-5, IL-6, and IL-13 in BALF and IL-4 in serum. Therefore, we speculate that FUT, FOY, and UTI decrease eosinophil infiltration, AHR, and airway remodeling by modulating the production and/or activity of Th2 cytokines.

Elevated IL-17 levels are found in BALF and blood of patients with severe asthma [27, 28]. IL-17 plays a critical role in airway inflammatory, neutrophil recruitment, airway hyperresponsiveness, and airway remodeling [29, 30] and enhances Th2 cell-mediated eosinophilic airway inflammation in asthma by regulating the expression of various inflammatory mediators [31]. IL-6 also plays a critical role in altering the balance between Treg and Th17 cells, and controlling IL-6 activity may be an effective approach for treating various autoimmune and inflammatory diseases [32]. In our study, serine protease inhibitors decreased the secretion of IL-6 and IL-17 in BALF and decreased neutrophil recruitment in the lungs, indicating that FUT, FOY, and UTI may inhibit neutrophil recruitment by modulating Th17 cell activity.

Corticosteroids and aspirin have long been used to treat inflammatory conditions. Mild and moderate asthma may be related to eosinophilic inflammation, which involves Th2 cell immune responses. However, severe asthma is corticosteroid resistance associated with neutrophilic inflammation, which is related to Th17 cell immune responses [33]. Patients with asthma who consume corticosteroids for a long time can develop serious side effects. Aspirin can inhibit Th17-associated inflammation, but it enhances Th2-associated inflammation in the airway [34]. In this study, serine protease inhibitors appeared to attenuate both Th2- and Th17-associated inflammation, which may contribute to therapy for severe asthma.

Previous studies demonstrated increased NF-*κ*B activity in allergic sensitization, which could also be induced by various agents such as ROS [3, 35]. NF-*κ*B, a redox-regulated transcription factor, plays a critical role in Th2 cell differentiation and regulates the production of proinflammatory mediators required for inducing allergic airway inflammation in pulmonary epithelial cells [36]. Therefore, the NF-*κ*B signaling pathway has been proposed as a promising target for therapeutic intervention in asthma [37]. In this study, FUT, FOY, and UTI inhibited NF-*κ*B activation in lung tissues as antioxidants.

It is worth noting that UTI, a urine trypsin inhibitor purified and concentrated from a human source, had a good effect on our repetitive Der p-challenged mouse model of chronic asthma. In our previous reports, UTI had no effect on a short-term sensitized murine model of asthma and may only be a biomarker of asthma exacerbation. The difference between these two studies was that the current study used long-term treatment in a repetitively Der p-challenged mouse model of chronic asthma. These results indicate that UTI may be an inflammatory biomarker associated with acute asthma exacerbation and have a feedback effect for improving airway inflammation in chronic allergic asthma. Detailed mechanisms underlying the effects of UTI on allergen-induced airway inflammation require further investigation.

## 5. Conclusions

In summary, our results demonstrated that the serine protease inhibitors FUT, FOY, and UTI attenuated AHR, allergic airway inflammation, and remodeling in a murine model of chronic asthma. These effects were associated with downregulation of Th2 cytokines and Th17 cell function and inhibition of NF-*κ*B activation in lung tissue. These serine protease inhibitors may have therapeutic benefits for treating asthma-associated airway inflammation.
